# Assessment of TVT and STS Risk Score Performances in Patients Undergoing Transcatheter Aortic Valve Replacement

**DOI:** 10.1016/j.jscai.2023.100600

**Published:** 2023-04-21

**Authors:** Karim Al-Azizi, Emily Shih, J. Michael DiMaio, John J. Squiers, Ghadi Moubarak, Austin Kluis, Jasjit K. Banwait, William H. Ryan, Molly I. Szerlip, Srinivasa P. Potluri, Mohanad Hamandi, Allison T. Lanfear, Talia G. Meidan, Robert C. Stoler, Timothy A. Mixon, Anita R. Krueger, Michael J. Mack

**Affiliations:** a Department of Cardiology, Baylor Scott & White The Heart Hospital, Plano, Texas; b Department of Cardiothoracic Surgery, Baylor Scott & White The Heart Hospital, Plano, Texas; c Baylor Scott & White Research Institute, Plano, Texas; d Department of Cardiology, Baylor University Medical Center, Dallas, Texas; e Department of Cardiology, Baylor Scott & White Medical Center–Temple, Temple, Texas; f Department of Cardiothoracic Surgery, Baylor Scott & White All Saints Medical Center, Fort Worth, Texas

**Keywords:** aortic stenosis, risk score, transcatheter aortic valve replacement

## Abstract

**Background:**

The Society of Thoracic Surgeons (STS) score has been used to risk stratify patients undergoing transcatheter aortic valve replacement (TAVR). The Transcatheter Valve Therapy (TVT) score was developed to predict in-hospital mortality in high/prohibitive-risk patients. Its performance in low and intermediate-risk patients is unknown. We sought to compare TVT and STS scores’ ability to predict clinical outcomes in all-surgical-risk patients undergoing TAVR.

**Methods:**

Consecutive patients undergoing TAVR from 2012-2020 within a large health care system were retrospectively reviewed and stratified by STS risk score. Predictive abilities of TVT and STS scores were compared using observed-to-expected mortality ratios (O:E) and area under the receiver operating characteristics curves (AUCs) for 30-day and 1-year mortality.

**Results:**

We assessed a total of 3270 patients (mean age 79 ± 9 years, 45% female), including 191 (5.8%) low-risk, 1093 (33.4%) intermediate-risk, 1584 (48.4%) high-risk, and 402 (5.8%) inoperable. Mean TVT and STS scores were 3.5% ± 2.0% and 6.1% ± 4.3%, respectively. Observed 30-day and 1-year mortality were 2.8% (92/3270; O:E TVT 0.8 ± 0.16 vs STS 0.46 ± 0.09), and 13.2% (432/3270), respectively. In the all-comers population, both TVT and STS risk scores showed poor prediction of 30-day (AUC: TVT 0.68 [0.62-0.74] vs STS 0.64 [0.58-0.70]), and 1-year (AUC: TVT 0.65 [0.62-0.58] vs STS 0.65 [0.62-0.58]) mortality. After stratifying by surgical risk, discrimination of the TVT and STS scores remained poor in all categories at 30 days and 1 year.

**Conclusions:**

An updated TAVR risk score with improved predictive ability across all-surgical-risk categories should be developed based on a larger national registry.

## Highlights

• The 30-day predictive ability of the transcatheter valve therapy and Society of Thoracic Surgeons scores was poor in all categories.
• The 1-year predictive ability of the transcatheter valve therapy and Society of Thoracic Surgeons scores was poor in all categories.
• An updated transcatheter aortic valve replacement risk score should be developed with improved predictive.

## Central Illustration

**Figure:**
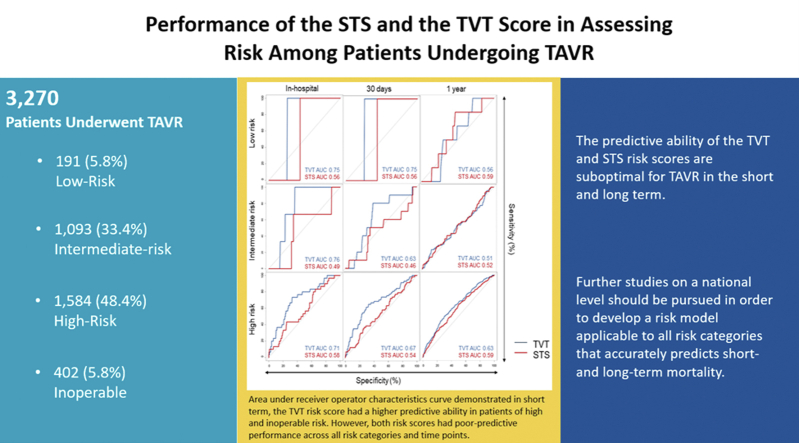
Performance of the STS and TVT score in assessing risk among patients undergoing TAVR.

## Introduction

Transcatheter aortic valve replacement (TAVR) has emerged as an effective treatment option for patients with severe aortic stenosis (AS) as an alternative to surgical aortic valve replacement (SAVR).^1^, ^2^, ^3^ Patients undergoing aortic valve replacement for severe AS often have multiple comorbidities, which increases perioperative morbidity and mortality and reduces late survival.^4^ The PARTNER trials demonstrated a significant reduction in mortality among high-risk patients undergoing TAVR compared with SAVR. However, overall mortality after TAVR in this particular group of patients remains relatively high.^5^ In contrast, patients with AS who were at intermediate or low surgical risk did not demonstrate significant differences in long-term mortality after TAVR compared with SAVR.^6^, ^7^ Therefore, accurate risk assessment and predictive tools are needed to inform physicians appropriately, counsel patients, and optimize the allocation of health care resources.

In the absence of a specific risk score for stratifying TAVR patients, the Society of Thoracic Surgeons (STS) Predicted Risk of Mortality (STS-PROM) score and the overall judgment of the site heart team were routinely used in clinical trials to classify TAVR patients with symptomatic severe AS. Moreover, The American Heart Association/American College of Cardiology (ACC) guidelines currently recommends assessing TAVR perioperative risk using only the STS-PROM score.^1^ However, when used to predict mortality after TAVR, this score has been shown to overestimate 30-day mortality. This is likely because the STS-PROM score was designed to predict outcomes among surgical patients; therefore, extrapolation to patients with TAVR remains challenging, and its appropriateness is controversial.

The STS/ACC Transcatheter Valve Therapy (TVT) Registry was created in 2011 to capture patient characteristics, procedural variables, and outcomes to report baseline benchmarks for performance of TAVR in the United States.^8^ A TAVR-specific risk model for in-hospital mortality was developed from data captured during the first 5 years of collection in the TVT registry.^9^, ^10^, ^11^, ^12^ However, TAVR was only commercially available to patients considered to be inoperable or at high surgical risk before 2016, and thus, the population from which the TVT score was derived did not include low and intermediate surgical risk patients undergoing TAVR. Although the TVT score has been validated in patients with high in-hospital mortality risk as predicted by STS-PROM,^13^ this score has not been validated for short-term outcomes in lower surgical risk populations nor for long-term outcomes in any risk population.

The goal of this study was to assess the predictive ability of the TVT risk model for short- and long-term mortality compared with the STS-PROM in patients of all-risk profiles undergoing TAVR.

## Methods

Consecutive patients undergoing TAVR between January 2012 and October 2020 within the Baylor Scott & White Health system were identified and analyzed. There were 4 participating hospitals performing TAVR within this large collaborative system. Center experience ranged from >10 years to <2 years. Patients were evaluated by an institutional multidisciplinary heart team consisting of cardiothoracic surgeons and cardiologists for eligibility for aortic valve intervention, including TAVR. Patients underwent formal preoperative evaluation and imaging through a TAVR clinic according to best practice guidelines established in our system. One-year follow-up information on survival status was collected using a previously validated protocol utilizing electronic medical record-confirmed contact with our health care system, patient phone calls, and online obituary searches.^14^ Patients were stratified into surgical risk groups according to the 2008 STS risk model^15^ (for cases from 2012 to 2017) and 2018 STS risk model^16^ (for cases from 2018 to 2020), which were defined as low-risk (STS-PROM <4%), intermediate-risk (STS-PROM ≥4%), high-risk (STS-PROM ≥8%), and inoperable (STS-PROM >15%) groups.

**Figure 1:**
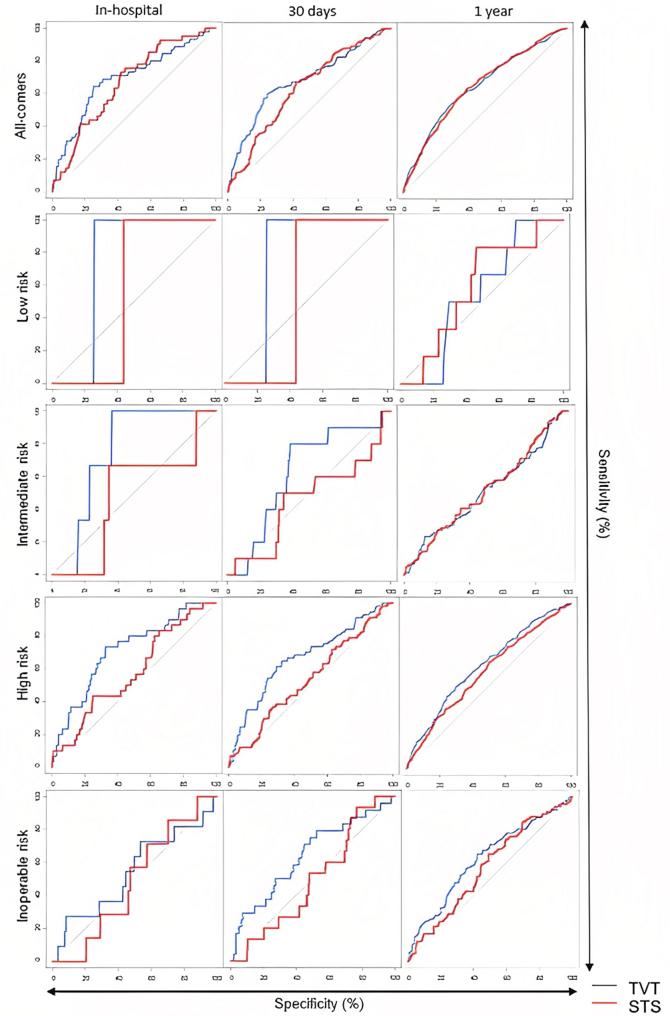
**Receiver operating characteristic curve for TVT and STS-PROM scores.** The area under receiver operator characteristics curves was utilized to determine predictive ability of the TVT and STS risk scores across all-risk categories. It demonstrated in short term, the TVT risk score had a higher predictive ability in patients of high and inoperable risk. However, both risk scores had poor-predictive performance across all-risk categories and time point. PROM, predicted risk of mortality; STS, Society of Thoracic Surgeons; TVT, transcatheter valve therapy.

Continuous variables are presented as mean ± standard deviation or median (interquartile range) as appropriate and categorical variables as proportions, unless otherwise specified. The predictive ability of TVT score and STS-PROM in predicting mortality at 30 days and 1 year was evaluated using area under the receiver operating characteristics curve (AUC) (Figure 1). While acknowledging that the STS score was initially designed to predict 30-day outcomes, we wanted to assess whether it could also predict 1-year all-cause mortality. An AUC >0.7 was considered acceptable predictive ability.^17^ Stratified bootstrapping technique was used to compute 95% confidence intervals with 2000 bootstrap replicates. In addition, the performance of the existing risk scores was evaluated using the observed-to-expected ratios (O:E) in predicting both short- and long-term mortality. The analysis was performed on both the all-comers study population and after stratification into surgical risk groups by STS-PROM as defined previously. All inferential and descriptive analyses were performed at the .05 significance level for a 2-sided test by using R version 4.0.0 (R Foundation for Statistical Computing).

The review board and ethics committee at Baylor The Heart Hospital - Plano approved this study. Given the retrospective nature of the study, informed consent was not required.

## Results

A total of 3270 patients underwent TAVR from 2012 to 2020 in a large health care system, ranging from 1733 procedures in the highest volume program to 70 in the lowest volume, newer program. Of these 3270 patients, 191 (5.8%) were low-risk, 1093 (33.4%) were intermediate-risk, 1584 (48.4%) were high-risk, and 402 (5.8%) were inoperable. The mean age was 79.2 ± 8.9 years, and 44.7% (1463/3270) of patients were female. At baseline, 68.2% (2223/3270) were classified as New York Heart Association classes III or IV, 89.9% (2940/3270) had hypertension, 22.6% (736/3270) had peripheral artery disease, and 40.1% (1308/3270) had diabetes. In addition, 15.7% (512/3270) had a prior permanent pacemaker implanted, 41.3% (1345/3270) had a prior percutaneous coronary intervention, and 23.6% (770/3270) had undergone coronary artery bypass grafting. Mean TVT score and STS-PROM were 3.5% ± 2.0% and 6.1% ± 4.3%, respectively (Table 1). As expected, mean TVT risk score increased with STS-PROM based surgical risk.

**Table 1:** Patient characteristics and outcomes.

Variable	Total (N = 3270)	Inoperable risk (n = 402)	High risk (n = 1584)	Intermediate risk (n = 1093)	Low risk (n = 191)	*P*
Age, y	79.2 ± 8.9	81.1 ± 9.4	80.1 ± 9.0	77.5 ± 8.6	73.3 ± 6.4	< 0.01
Female	1463 (44.7)	203 (50.5)	748 (47.2)	453 (41.4)	59 (30.9)	< 0.01
BMI, kg/m^2^	29.3 ± 13.3	28.1 ± 10.57	28.5 ± 10.0	30.4 ± 16.7	32.3 ± 18.3	< 0.01
Hypertension	2940 (89.9)	360 (89.6)	1417 (89.5)	1006 (92.0)	157 (82.2)	< 0.01
Smoker	211 (6.5)	28 (7.0)	93 (5.9)	80 (7.4)	10 (5.2)	0.40
PAD	736 (22.6)	126 (31.4)	405 (25.7)	187 (17.1)	18 (9.4)	< 0.01
Diabetes	1308 (40.1)	172 (42.9)	666 (42.2)	412 (37.7)	58 (30.7)	< 0.01
Chronic lung disease	1252 (38.3)	205 (51.0)	670 (42.3)	344 (31.5)	33 (17.3)	< 0.01
NYHA class III-IV	2223 (68.2)	326 (81.5)	1182 (74.9)	656 (60.1)	59 (30.8)	< 0.01
Prior PPM	512 (15.7)	82 (20.4)	306 (19.4)	111 (10.2)	13 (6.8)	< 0.01
Prior ICD	140 (4.3)	22 (5.5)	80 (5.1)	35 (3.2)	3 (1.6)	0.02
Prior PCI	1345 (41.3)	167 (41.9)	680 (43.1)	448 (41.1)	50 (26.5)	< 0.01
Prior CABG	770 (23.6)	127 (31.7)	427 (27.0)	206 (18.9)	10 (5.3)	< 0.01
TVT score	3.5 ± 2.0	4.8 ± 2.9	3.9 ± 2.0	2.8 ± 1.2	2.1 ± 0.6	< 0.01
STS-PROM score	6.1 ± 4.3	10.2 ± 5.6	7.5 ± 4.2	3.9 ± 1.7	1.6 ± 0.7	< 0.01

Values are presented as number (%) or mean ± standard deviation. Demographics and comorbidities characterizing patients undergoing TAVR and stratified by surgical risk based on the calculated STS-PROM. Most patients fell into the high-risk surgical category given the timeframe of the study.

BMI, body mass index; CABG, coronary artery bypass grafting; ICD, implantable cardiac defibrillator; NYHA, New York Heart Association; PAD, peripheral artery disease; PCI, percutaneous coronary intervention; PPM, permanent pacemaker; STS-PROM, Society of Thoracic Surgeons Predicted Risk of Mortality; TAVR, transcatheter aortic valve replacement; TVT, transcatheter valve therapy.

Overall mortality was 2.8% (92/3270) at 30 days and 13.2% (432/3270) at 1 year. Observed mortality within each risk category as well as the observed-to-expected (O:E) ratios based on the TVT risk score and STS-PROM are detailed in Tables 2 and 3, respectively. Overall, the observed short-term mortality was closer to the expected mortality based on TVT risk score. Both scores overestimated the mortality risk with an exception of the TVT risk score at 30 days in patients with inoperable risk (O:E 1.24 ± 0.5). At 1 year, both risk scores grossly underestimated the mortality risk. The mortality rate at each time point increased with the severity of the surgical risk categorization. Both TVT score and STS-PROM performed poorly at 30 days and 1 year for all-risk patients and when subjected to surgical risk stratification (Table 4). After risk stratification, the TVT score performed better than the STS-PROM at both 30 days and 1 year in high-risk and inoperative patients only (Table 4).

**Table 2:** Outcomes and predictability of the TVT risk score.

Variable	Total (N = 3270)	Inoperable risk (n = 402)	High risk (n = 1584)	Intermediate risk (n = 1093)	Low risk (n = 191)
In-hospital mortality	45 (1.4)	11 (2.7)	30 (1.9)	3 (0.3)	1 (0.5)
O	1.4	2.7	1.9	0.3	0.5
E	3.5	4.8	3.9	2.8	2.1
O:E mortality	0.39 ± 0.11	0.57 ± 0.34	0.49 ± 0.17	0.1 ± 0.11	0.25 ± 0.49
30-d mortality	92 (2.8)	24 (6.0)	57 (3.6)	10 (0.9)	1 (0.5)
O	2.8	6	3.6	0.9	0.5
E	3.5	4.8	3.9	2.8	2.1
O:E mortality	0.8 ± 0.16	1.24 ± 0.5	0.92 ± 0.24	0.33 ± 0.2	0.25 ± 0.49
1-y mortality	432 (13.2)	86 (21.4)	264 (16.7)	76 (7.0)	6 (3.1)
O	13.2	21.4	16.7	7	3.1
E	3.5	4.8	3.9	2.8	2.1
O:E mortality	3.77 ± 0.36	4.46 ± 0.94	4.27 ± 0.52	2.48 ± 0.56	1.5 ± 1.2

Values are presented as n (%). Observed-to-expected mortality [O:E] presented as ratio ± standard error. The O:E ratio is calculated based off the TVT risk score for in-hospital mortality across each surgical risk category.

E, expected; O, observed; TVT, transcatheter valve therapy.

**Table 3:** Outcomes and predictability of the STS risk score.

Variable	Total (N = 3270)	Inoperable risk (n = 402)	High risk (n = 1584)	Intermediate risk (n = 1093)	Low risk (n = 191)
In-hospital mortality	45 (1.4)	11 (2.7)	30 (1.9)	3 (0.3)	1 (0.5)
O	1.4	2.7	1.9	0.3	0.5
E	6.1	10.2	7.5	3.9	1.6
O:E mortality	0.23 ± 0.07	0.27 ± 0.16	0.25 ± 0.09	0.07 ± 0.08	0.33 ± 0.64
30-day mortality	92 (2.8)	24 (6.0)	57 (3.6)	10 (0.9)	1 (0.5)
O	2.8	6	3.6	0.9	0.5
E	6.1	10.2	7.5	3.9	1.6
O:E mortality	0.46 ± 0.09	0.59 ± 0.23	0.48 ± 0.12	0.23 ± 0.15	0.33 ± 0.64
1-year mortality	432 (13.2)	86 (21.4)	264 (16.7)	76 (7.0)	6 (3.1)
O	13.2	21.4	16.7	7	3.1
E	6.1	10.2	7.5	3.9	1.6
O:E mortality	2.17 ± 0.2	2.1 ± 0.44	2.22 ± 0.27	1.78 ± 0.4	1.96 ± 1.57

Values are presented as n (%). Observed-to-expected mortality [O:E] presented as ratio ± standard error. The O:E ratio is calculated based off the STS risk score for 30-day mortality across each surgical risk category. E, expected. O, observed; STS, Society of Thoracic Surgeons.

**Table 4:** Predictability of TVT and STS scores stratified by risk category.

Risk category	AUC	Bootstrapped 95% CI
In-hospital		
All comers		
TVT score	0.69	0.61-0.78
STS-PROM	0.67	0.60-0.74
Low risk		
TVT score	0.75	0.43-1
STS-PROM	0.56	0.2-0.8
Intermediate risk		
TVT score	0.76	0.65-0.85
STS-PROM	0.49	0.13-0.69
High risk		
TVT score	0.71	0.62-0.80
STS-PROM	0.58	0.49-0.68
Inoperable risk		
TVT Score	0.55	0.37-0.72
STS-PROM	0.49	0.32-0.65
30-day		
All comers		
TVT score	0.68	0.62-0.74
STS-PROM	0.64	0.58-0.70
Low risk		
TVT score	0.75	0.43-1
STS-PROM	0.56	0.2-0.8
Intermediate risk		
TVT score	0.63	0.47-0.76
STS-PROM	0.46	0.26-0.65
High risk		
TVT score	0.67	0.59-0.75
STS-PROM	0.54	0.47-0.62
Inoperable risk		
TVT Score	0.64	0.52-0.75
STS-PROM	0.50	0.37-0.62
1-year		
All comers		
TVT score	0.65	0.62-0.68
STS-PROM	0.65	0.62-0.68
Low risk		
TVT score	0.56	0.40-0.71
STS-PROM	0.59	0.41-0.77
Intermediate risk		
TVT score	0.51	0.44-0.59
STS-PROM	0.52	0.45-0.59
High risk		
TVT score	0.63	0.59-0.66
STS-PROM score	0.59	0.55-0.63
Inoperable risk		
TVT Score	0.62	0.55-0.69
STS-PROM	0.57	0.49-0.65

AUC curves for the TVT and STS-PROM across all-risk categories. An AUC >0.7 was considered acceptable predictive ability. AUC, area under the curve; CI, confidence interval; STS-PROM, Society of Thoracic Surgeons Predicted Risk of Mortality; TVT, transcatheter valve therapy.

## Discussion

Advances in the technique of aortic valve replacement and the periprocedural phases of care have reduced operative risk to low levels, even among high-risk patients. However, some fatal complications remain unpredictable and unavoidable. The accuracy of risk-stratifying tools and clinical prediction models used to guide clinical decision-making and interventions should be validated before broad clinical implementation.^18^ In the absence of appropriate paradigms for risk stratification, calculation of the STS score remains the recommended method for assessing perioperative risk in TAVR. However, the extension of its use to predict clinical outcomes among the cohort of TAVR patients is controversial. Indeed, the derivation of the STS score was based on a dataset composed only of surgical patients and was therefore not designed to predict mortality in patients undergoing TAVR. The STS/ACC TVT risk model was developed to predict in-hospital mortality after TAVR. Notably, the TVT risk score was developed at a time when TAVR patients were, predominantly, at high or prohibitive surgical risk.^13^ Since then, the use of TAVR has expanded to include patients across the full spectrum of surgical risk.

The TVT registry captures patient data specific to TAVR patients, so it is pertinent to evaluate the predictive performance of the TVT risk model against STS-PROM in patients of all-risk categories. It is also essential to understand the predictive ability of these scores beyond the index hospital stay or first 30 days after TAVR. In this study, we found that the TVT risk model was not a more accurate predictor of mortality than the STS-PROM in low and intermediate-risk patients, although it did have improved predictive ability in high-risk and inoperable patients. However, the predictive performance of both TVT and STS risk scores were suboptimal in patients of all-risk categories and time points overall (Central Illustration).

**Central Illustration:**
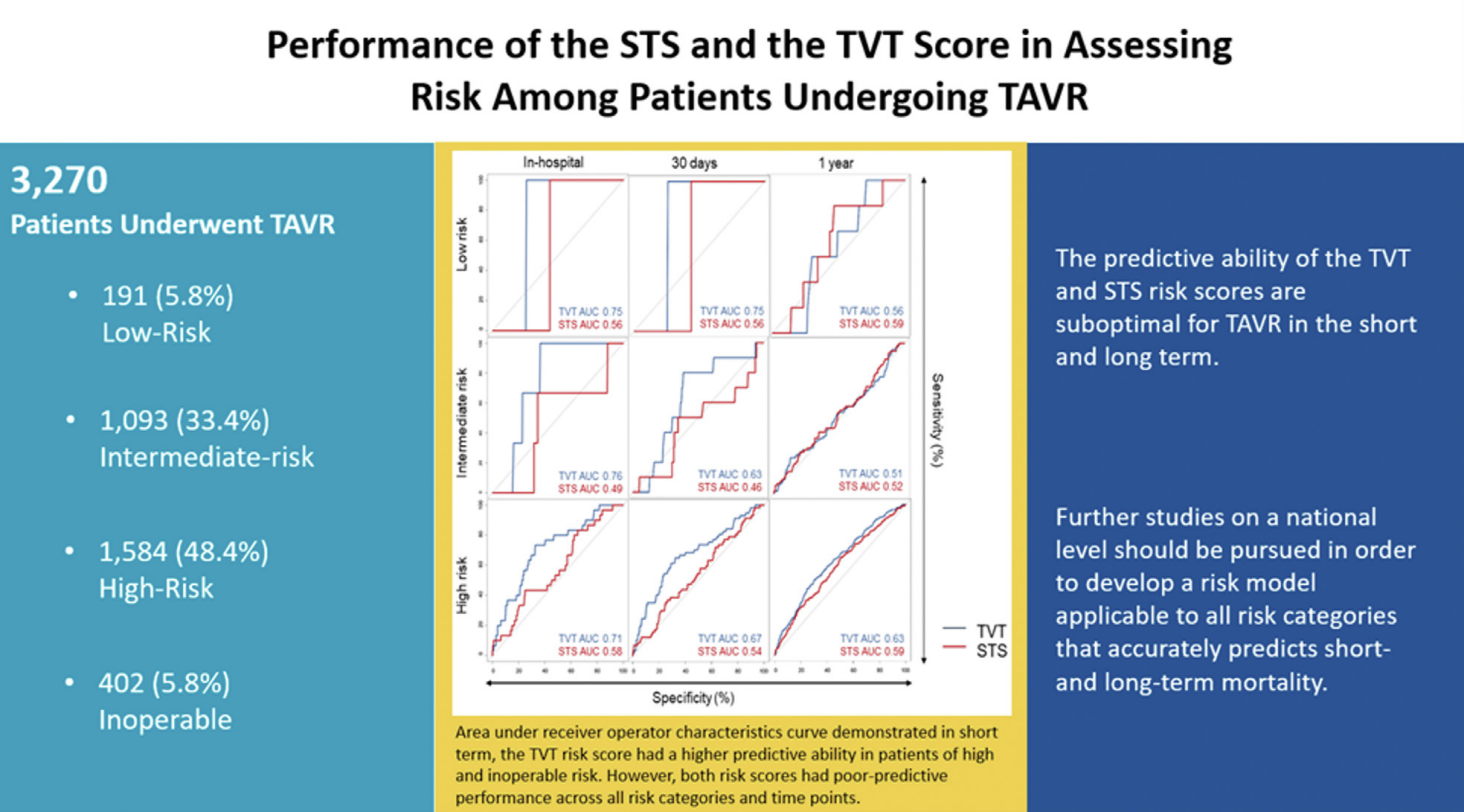
Performance of the STS and TVT score in assessing risk among patients undergoing TAVR.

### Performance of TVT and STS risk score in all-comers population

In the era of increasing TAVR procedures, TAVR-specific risk scores derived from multiple databases have been developed worldwide. In our study, the predictive ability of the TVT risk score was essentially equivalent to that of the STS risk score within the all-comers population in the short term. Both scores overestimated mortality risk based on our observed outcomes. Other studies have yielded differing results. An analysis assessing the performance of surgical risk scores (Euroscore II and STS) and TAVR risk scores (German Aortic Valve score, FRANCE-2 score, OBSERVANT model, and the TVT risk model) against the United Kingdom Transcatheter Aortic Valve Implantation (UK TAVI) registry found that the TVT risk score had a better predictive performance at 30 days compared with the surgically derived scores. In addition, this study showed that TAVR-specific scores had higher discrimination than surgical risk scores.^19^ Even so, the predictive performance of all-risk categories in this study was marginally good (AUC <0.7). An improved risk score for patients of all-risk categories undergoing TAVR is therefore necessary.

Despite some hesitant optimism for the performance of the TVT score up to 30 days, the applicability of the TVT risk model beyond the short term has not been widely evaluated. Our long-term findings suggest that both risk scoring systems have poor-predictive power. Therefore, to guide patient selection, newer prediction models with better discrimination and calibration tools should be developed, not only for short-term but also for longer-term follow-up.

### Performance of TVT and STS risk score in high-risk patients

The inconsistency between the observed and expected 30-day mortality with cardiac surgery risk scores in TAVR patients was first highlighted in the PARTNER cohort A trial^4^ and reinforced by other studies.^20^, ^21^, ^22^ The TVT score was derived from high-risk patients undergoing TAVR and was designed to predict in-hospital mortality,^13^ whereas the STS score was derived from all-comers data for patients undergoing SAVR. Compared with the STS score, the TVT risk model includes more limited and objective parameters for calculating risk of mortality compared with the STS-PROM to minimize interobserver variability and potentially improve predictive ability.^23^ It is not surprising, therefore, that our data show that in the short term, the TVT score outperformed the STS score in high-risk patients, with more favorable observed-to-expected ratios. This finding is consistent with previous validation studies.^9^ Additionally, we found that the TVT score moderately outperformed the STS-PROM up to 1 year from TAVR in patients of high or inoperable risk.

The TVT and STS-PROM scores do not collect quality-of-life data that can serve as a general index of procedural benefit, especially among high-risk patients. An example of this could be The Kansas City Cardiomyopathy Questionnaire, assessed at 30 days and 1 year after the procedure. Therefore, linking this kind of information to the wealth of clinical data in the TVT and STS scores will allow models to be built to predict the probability of TAVR benefit. For example, a patient may have a good prediction of TAVR mortality but a low probability of procedural benefit. Information of this type should be considered when designing new risk models and should serve as powerful tools in selecting appropriate patients.

### Performance of TVT and STS-PROM in low- and intermediate-risk patients

The applicability of the TVT score in low- and intermediate-risk patients undergoing TAVR has not been well studied. Our findings show that the STS score performs poorly (AUC <0.6) in the intermediate-risk population. The observed mortality was much lower than that predicted by TVT and STS scores. Our findings are consistent with previously published studies demonstrating that STS-PROM overpredicts postoperative mortality in intermediate- and low-risk patients.^24^^,^^25^ In addition, the TVT score did not have greater predictive power than STS-PROM in either the short or long term. This suggests that there is currently no adequate predictive model for intermediate-risk patients undergoing TAVR. Regarding the low-risk population, the small percentage (5.8%) of low-risk patients in our study makes it difficult to draw firm conclusions for this patient group, underscoring the need for future studies with a larger sample of low-risk patients to validate or refute existing risk scores.

### Future direction of prediction models for short- and long-term mortality

Although the higher predictive ability of the TVT risk model compared with the STS-PROM in high-risk patients is reinforced in our study, the current risk models developed from TAVR-specific registries are suboptimal across all-risk groups. In fact, both risk scores have obvious limitations; patients can have a very high operative mortality risk and still have low scores. There are several comorbidities not captured in the STS and TVT scores, such as degree of vascular calcification, porcelain aorta, chest wall abnormalities, frailty, immunosuppression, cirrhosis, and others. Frailty is particularly difficult to quantify. External validation studies using the Israeli TAVI registry,^26^ and Swiss TAVI registry^12^ (C-statistics were 0.66 and 0.68 for the TVT risk model, respectively) have repeatedly demonstrated, at best, moderate predictive performance of the TVT score. Attempts to update the TVT risk model based on contemporary patient data to account for calibration shift have not been largely successful (AUC 0.6 to 0.63).^27^ Given the increasingly widespread use of the TAVR procedure and the ability to capture patient and procedural details within the TVT registry, the development of an improved risk score is needed. Data from the TVT registry on a national level is needed to recalibrate the existing model or to create an entirely new risk score that is more accurate in the short and long term, and applies to patients of all-risk categories who are candidates for TAVR.

### Limitations

This study is subject to all limitations present in retrospective, observational studies. This information was pooled from a large regional health care system, therefore, the generalizability to a larger TAVR population with differing operator experience and beyond our geographical region is uncertain. Another limitation is the variability in cohort size of patients within the different risk categories. There were significantly fewer patients in the low-risk category, and lesser events (mortalities); therefore, it is difficult to make strong conclusions based on the wide confidence intervals in our resulting metrics. This is not surprising, however, as the majority of patients undergoing TAVR will fall into higher risk categories given the timeframe of the study. Future studies with a larger sample size of low-risk patients should be performed to validate the existing risk scores for both long- and short-term mortality.

## Conclusion

This study demonstrates that the predictive ability of the TVT and STS risk scores for TAVR is suboptimal in the short and long term. Comparatively, the TVT risk score is more predictive than the STS score only in patients with high surgical risk and inoperable patients. In patients with low and intermediate risk, both scores are poor discriminators of 30-day and 1-year mortality. Further studies on a national level should be conducted to develop a TAVR-specific risk model that applies to all-risk categories to optimize patient selection and improve clinical outcomes. Until such a validated TAVR risk score is developed, clinical judgment by the on-site heart team should be combined with conventional scores to guide clinical decision making.
